# Unveiling the Effects of Quicklime on the Properties of Sulfoaluminate Cement–Ordinary Portland Cement–Mineral Admixture Repairing Composites and Their Sulphate Resistance

**DOI:** 10.3390/ma16114026

**Published:** 2023-05-28

**Authors:** Chen Shi, Ying Yang

**Affiliations:** College of Materials Science and Engineering, Xi’an University of Architecture and Technology, Xi’an 710055, China; shichen@xauat.edu.cn

**Keywords:** repair material, quicklime, sulphoaluminate cement, sulphate attack, porosity

## Abstract

Aiming at the problem of repeated repair of concrete caused by the damage of concrete structure repair system in a sulphate environment, the quicklime modified sulphoaluminate cement (CSA)–ordinary Portland cement (OPC) –mineral admixture composite repair material was utilised to obtain the law and mechanism of quicklime, improving the mechanical properties and sulphate resistance of composite repair materials. In this paper, the effects of quicklime on the mechanical properties, as well as sulphate resistance of CSA–OPC–ground granulated blast furnace slag (SPB) and CSA–OPC–silica fume (SPF) composites, were studied. The findings reveal that the addition of quicklime improves the stability of ettringite in SPB and SPF composite systems, promotes the pozzolanic reaction of mineral admixtures in composite systems, and significantly increases the compressive strength of both SPB and SPF systems. The 8 h compressive strength of SPB and SPF composite systems increased by 154% and 107%, and the 28 d compressive strength enhanced by 32% and 40%. After the quicklime was added, the formation of C-S-H gel and calcium carbonate in SPB and SPF composite systems was promoted, the porosity was reduced, and the pore structure was refined. The porosity was reduced by 2.68% and 0.48%, respectively. The mass change rate of various composite systems under sulphate attack was reduced, and the mass change rate of the SPCB30 and SPCF9 composite systems decreased to 0.11% and −0.76% after 150 dry–wet cycles. Additionally, the mechanical strength of different composite systems under sulphate attack was improved, so that the sulphate resistance of different ground granulated blast furnace slag and silica fume composite systems was improved.

## 1. Introduction

Sulphate attack is a significant factor impacting the service life of concrete in the saline soil areas all over the world [1,2,3]. The environmental soil and groundwater in this area contain a large amount of sulphate, which will lead to damage problems, including expansion, cracking, and spalling of concrete [4,5]. Currently, the new concrete structures in this area have paid attention to this issue in recent years and adopted the concrete mix design of sulphate resistance, and most of the engineering treatment has selected new technology, along with new material technology [6]. Zhao [7] designed an erosion resistant and durable C50 concrete mix. This ratio was formally constructed in the Salt Lake section. In the Yili area of Xinjiang, the hydraulic concrete ratio of sulphate-resistant cement is formulated to resist sulphate erosion, and the effect of sulphate erosion is good [8]. However, a few existing concrete structures have been eroded by saline soil environment, and the amount of work needed to be repaired is huge. Concerning repair materials in sulphate corrosion environments, there are not many reports. Repairing with ordinary repair mortar in a sulphate corrosion environment often leads to secondary spalling within a short period of time, resulting in a waste of manpower and material resources [9,10]. Therefore, designing a cement-based rapid repair material resistant to sulphate corrosion, prolonging the service life of the repair system, and reducing the repair cost is of great significance [11,12].

CSA (sulphoaluminate cement) has been widely used in many rapid repair projects because of its fast setting and hardening, high early strength, and good corrosion resistance [13,14]. The later performance of this cement is nevertheless unstable and limited in use [15,16]. Relevant research indicates that [17,18] stabilising the development of later strength can be achieved by adding an appropriate amount of OPC (ordinary Portland cement) to CSA. Therefore, the CSA–OPC composite system is often used in the repair of concrete buildings in sulphate areas and the anti-corrosion of marine concrete buildings [15]. However, the effect is still not ideal after using this material for repairing concrete projects with severe sulphate corrosion [19,20].

The incorporation of mineral admixtures into OPC, according to previous studies, can significantly improve the later strength and durability of cement-based materials, such as impermeability, frost resistance, and sulphate corrosion resistance [21,22]. Due to the low alkalinity of CSA, mineral admixtures cannot be effectively employed in sulphoaluminate cement-based materials, and they only play a filling role [23]. Even though some studies have suggested that mineral admixtures might enhance the pore structure of CSA [24], the ettringite in the CSA system is easily decomposed in low alkalinity environments, and the pozzolanic activity of admixtures is difficult to be stimulated in these conditions [25].

Ding et al. [26] studied that ground granulated blast furnace slag, fly ash, and silica fume can improve the sulphate resistance of the CSA–OPC composite system to varying degrees, but the incorporation of mineral admixtures will decrease the mechanical properties of mortar to varying degrees. The method of combining a composite salt-dry–wet cycle with semi-immersion was used by Zhang et al. [27] to simulate the saline soil erosion environment. It was concluded that nano-SiO_2_ and appropriate slag could improve the compactness of mortar and enhance the corrosion resistance of the CSA–OPC composite repair mortar to saline soil. Atahan et al. [28] claimed that both ground granulated blast furnace slag and nano-SiO_2_ considerably reduced the expansion caused by sulphate attack and enhanced the resistance to it, but only a small amount of 2% nano-SiO_2_ could improve the resistance to sulphate attack of the composite system. As a result, the components of repair materials for saline soil environments should be designed.

Quicklime is abundant, easy to obtain, and relatively inexpensive, and its alkalinity will not only stimulate the mineral admixtures in the repair system, but likewise improve the overall alkalinity of the repair system and offers a good alkaline environment for the stable existence of the main hydration product ettringite in the repair system [29,30]. However, the sulphate corrosion performance of quicklime on composite systems is not yet clear.

As a result, the effects of quicklime on the setting time, mechanical properties, and sulphate resistance of CSA–OPC–ground granulated blast furnace slag (SPB) and CSA–OPC–silica fume (SPF) composite systems were systematically examined in this paper. The hydration products and micro-morphology were analysed using X-ray diffractometry, thermogravimetric, scanning electron microscopy, and mercury intrusion. A thorough explanation of the influence mechanism of quicklime on its mechanical properties and sulphate resistance was given. In addition to providing a feasible design ratio of repair materials for concrete repair projects affected by sulphate erosion, it also offers a theoretical basis for the engineering application of quicklime modified CSA–OPC–mineral admixture repair system in a sulphate erosion environment.

## 2. Materials and Methods

### 2.1. Raw Materials

The raw materials were OPC type P·O 42.5, produced by Conch Cement Co., Ltd. produced in Xi’an, China and Shili CSA produced in Dengfeng City, China (CSA 42.5). The components of cement and mineral admixtures are analyzed using XRF. The chemical composition and physical properties of the OPC and CSA are listed in Table 1 and Table 2, respectively. The ground granulated blast furnace slag (GGBS) and silica fume (SF) was purchased from Gongyi Longze Water Purification Material Co., Ltd. produced in Tianjin. The chemical composition of the GGBS and SF are listed in Table 1. Quicklime produced by Tianjin Zhiyuan Chemical Reagent Co., Ltd. born in Zhengzhou, China (CaO content over 98%), and naphthalene superplasticizer (BNS) provided by Shaanxi Longsheng Building Materials Co., Ltd. produced in Xi’an, China Yellow powder form with a water reduction rate of 20%; the sand used was washed river sand from Hanzhong with a fineness modulus of 2.6. The test water is purified tap water in the laboratory.

### 2.2. Mixture Proportion of Composite Cement Slurry

**Figure 1:**
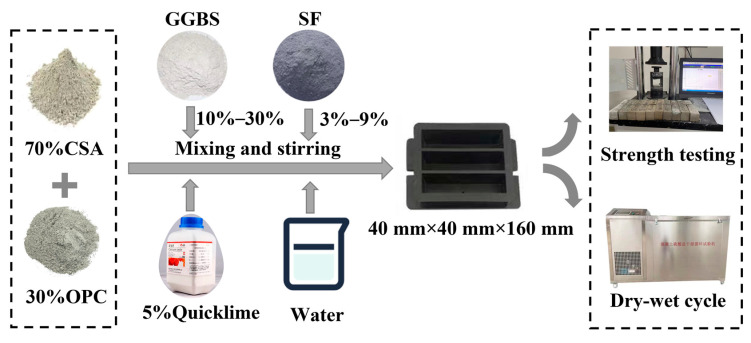
Specimen preparation flow chart.

In this experiment, mortar with cement–sand ratio of 1:1.5 and water–binder ratio of 0.3 was prepared. The test ratio of repair mortar is shown in Table 3, where SP is a CSA–OPC (7:3) benchmark system. SPB10, SPB20, and SPB30 and SPF3, SPF6, and SPF9 indicate that the cement rates of the CSA–OPC system replaced by the ground granulated blast furnace slag and silica fume are 10%, 20%, 30% and 3%, 6%, and 9% respectively. SPCB10, SPCB20, and SPCB30 and SPCF3, SPCF6, and SPCF9 indicate that 5% quicklime is added to the ratio of SPB10, SPB20, and SPB30 and SPCF3, SPCF6, and SPCF9 composite systems. The specimen preparation flow chart could be referred to Figure 1.

### 2.3. Experimental Methods

#### 2.3.1. Setting Time

The setting time of cement slurry was tested using a Vicat apparatus regarding the Chinese Standard-Test method for water consumption, setting time, and stability of cement standard consistency (GB/T 1346–2011). Both the initial setting time and the final setting time were recorded.

#### 2.3.2. Mechanical Strength

The strength specimens were molded into 40 mm × 40 mm × 160 mm specimens according to the standard of repair mortar (JC/T 2381-2016) and cured under standard conditions (temperature 20 ± 2 °C, relative humidity 90 ± 5%). The strength test method refers to the cement mortar strength test method (GB/T 11761-2011). The test specimens of 8 h, 1 d, 7 d, and 28 d were tested using a YAW-300 electronic universal mechanical testing machine with a loading rate of 0.6 MPa/s produced in Wuxi City, China. Three specimens of each mix proportion were used to determine the mean and standard deviation of the compressive strength.

#### 2.3.3. Sulphate Resistance Performance

The sulphate resistance performance was referred to the Cement Sulphate Resistance Test Method (GB/T 749-2008). The erosion solution was 5% Na_2_SO_4_. After 7 days of standard curing, the sample was placed in a concrete sulfate dry–wet cycle test box (soaking for 15 h, air drying for 1 h, drying at 80 °C for 6 h, cooling for 2 h, every 24 h is a cycle). The mass and strength after 10 times, 25 times, 40 times, 60 times, 90 times, 120 times, and 150 times of dry–wet cycle erosion were tested, respectively, and the mass change rate was calculated according to Formula (1).

(1) $$W_{1}=\frac{M_{n}-M_{0}}{M_{0}}\times 100\%$$

where: $W_{1}$—mass change rate (%); $M_{n}$—mass after erosion (g); $M_{0}$—mass before erosion (g).

#### 2.3.4. X-ray Diffractometry (XRD)

XRD analysis was performed to examine the mineral composition alteration of SP pastes due to water and Na_2_SO_4_ attacks. The fine powders were analyzed using a Shimadzu XRD 6100 X-ray diffractometer (XRD) produced in Japan. The samples were scanned from 5 to 60° (2θ) at a rate of 30 s/° with the step of 0.02°. The 40 mm × 40 mm × 40 mm external pastes surfaces of the specimens were ground to a depth of around 1–2 mm. The overall surfaces of the specimens were ground to remove the concentration of excessive sulphate on the surfaces. These plates were broken into pieces. To prevent their hydration, the broken fragments of the modified mortar sample were placed in a sample tube containing a mixed solution of ethanol and acetone. The sample with stopped hydration was then ground in a mortar, dried in a vacuum oven for 72 h, sealed, and stored for analysis and testing. Each powder sample was sieved through a 45 μm sieve to obtain cement powder. The cement powder samples after passing through the 45 μm sieve were assessed via XRD.

#### 2.3.5. Thermogravimetric Analysis (TG)

TG was performed on a Netzsch TG 209 F3 analyzer produced in Germany under nitrogen gas atmosphere, purged at 58 mL/min. About 16–18 mg of additionally ground powder sample was heated in a platinum crucible at a rate of 10 °C/min up to 800 °C. Thermogravimetric analysis can help analyze the type and number of products formed during the reaction of cement. Therefore, the samples were washed with acetone and soaked in acetone for 48 h to stop the hydration reaction between cement and water. Acetone, which was chosen because it can be used in mixed water, was used to extract water from the cement composite amorphous, thus preventing the hydration reaction between cement and water.

#### 2.3.6. Scanning Electron Microscopy (SEM)

The microstructure after 90 times of dry–wet cycle erosion were tested. The microstructure was tested by SEM ZEISS Sigma 300 produced in Germany. The microstructure and internal structure of the complex cement paste were observed by SEM analysis. The specimens collected from the compressive strength test were immersed in alcohol for 24 h, then dried in a vacuum drying oven, and stored under a vacuum for another 7 days. The samples were gold coated, and the observation was conducted under high vacuum with a voltage of 15 kV and a working distance of 10 mm.

#### 2.3.7. Mercury Intrusion Porosimetry (MIP)

The 30 d specimens collected from the compressive strength test were immersed in alcohol for 24 h, then dried in a vacuum drying oven, and stored under a vacuum for another 3 days. The mercury intrusion was tested by AutoPore 9500 produced in Shenzhen, China, which was used to obtain the porosity and pore size distribution of specimens.

## 3. Results

### 3.1. Effect of Quicklime on Setting Time of SPB and SPF Pastes

**Figure 2:**
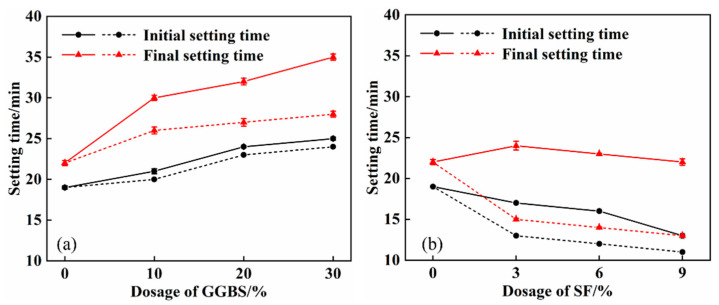
Effect of quicklime on setting time of composite system: (**a**) SPCB solid line, SPB dotted line; (**b**) SPCF solid line, SPF dotted line.

Figure 2 depicts the variation of the setting time of the composite system with the content of GGBS and SF. The dotted line denotes the setting time diagram of SPB and SPF, and the solid line represents the setting time diagram of SPCB and SPCF following the addition of quicklime. As highlighted in Figure 2, the setting time of the composite system increases as the GGBS content increases, and the setting time decreases as the SF content increases.

After adding quicklime, the setting time of different composite systems is prolonged. The initial setting time of SPCB and SPCF is 1 min and 3 min longer in comparison to that of SPB and SPF, respectively, and the final setting time is 5 min and 9 min longer than that of SPB and SPF, respectively. With 30% GGBS content, the final setting time of SPCB30 can reach 35 min. With the increase in GGBS content, the mineral composition and hydration products of cement are reduced, which prolongs the setting time [31]. SF is finer than cement particles, which promotes the nucleation of hydration products while accelerating the crystallisation of hydration products. Therefore, the setting time is shortened with the increase in SF content [32]. When quicklime is added to the composite system, the isomorphous effect of Ca^2+^ delays the hydration of C_3_A, and the calcium hydroxide produced by quicklime has an excitation effect on GGBS and SF. However, the reaction of active components in GGBS and SF with calcium hydroxide is very slow at room temperature, prolonging the setting time [33,34].

### 3.2. Effect of Quicklime on Mechanical Properties of SPB and SPF Repair Mortar

**Figure 3:**
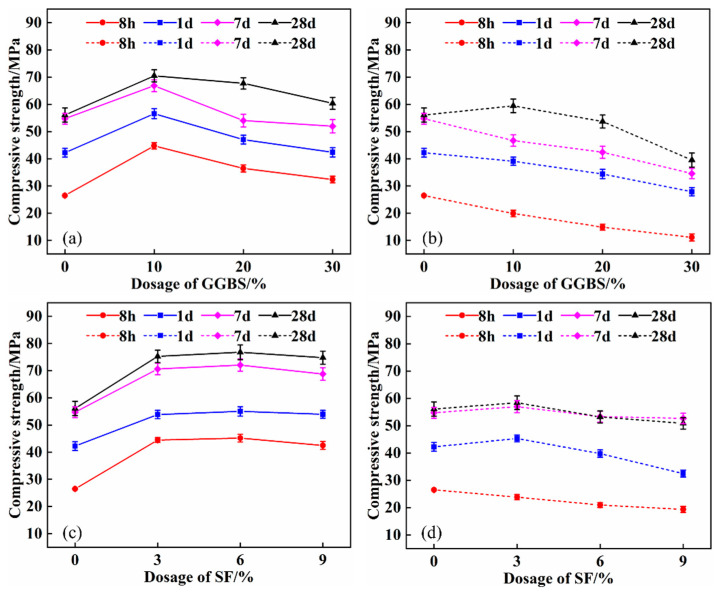
Effect of quicklime on compressive strength of composite system: (**a**) SPCB; (**b**) SPB; (**c**) SPCF; (**d**) SPF.

The change in compressive strength of the composite system with the content of GGBS and SF is presented in Figure 3. The dotted line reflects the compressive strength of SPB and SPF, while the solid line represents the compressive strength of SPCB and SPCF after the addition of quicklime. Figure 3 shows that the compressive strength of SPB and SPF composite systems decreases as the GGBS and SF content rises. Following the addition of 30% GGBS, the 8 h and 28 d compressive strength of the SPB30 composite system decreased by 58% and 30%, respectively, while the 8 h and 28 d compressive strength of the SPF9 composite system decreased by 27% and 9%, respectively, after adding 9% SF.

The compressive strength of the composite system was dramatically improved after the addition of quicklime. The 8 h and 28 d compressive strength of the SPCB30 composite system improved by 191% and 53%, respectively, when the GGBS content was 30%. When the SF content was 9%, the 8 h and 28 d compressive strength of the SPCF9 composite system improved by 120% and 47%, respectively. Cement is replaced with mineral admixtures in the SPB and SPF composite system, which lowers the early hydration reaction substances in the system. Simultaneously, because the CSA is a low alkalinity cement, when the admixture content increases, the hydration product CH is insufficient, and the admixture’s activity cannot be well stimulated, so the strength is reduced [35]. However, the addition of quicklime provides a suitable alkaline excitation environment for the system, which facilitates the formation of C-S-H gel from GGBS and SF, thereby improving the compressive strength [36,37].

**Figure 4:**
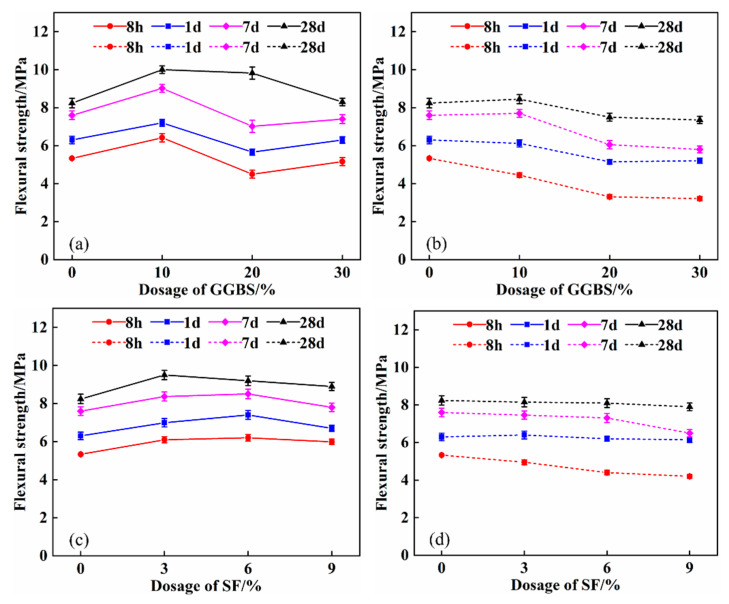
Effect of quicklime on flexural strength of composite system: (**a**) SPCB; (**b**) SPB; (**c**) SPCF; (**d**) SPF.

The variation of flexural strength of SPB and SPF with the content of GGBS and SF after incorporating quicklime is shown in Figure 4. Figure 4 shows that, as GGBS and SF content are increased, the flexural strength of SPB and SPF composite systems decreases. The flexural strength of the SPB30 composite system is the lowest, and the flexural strength of 8 h and 28 d is 3.2 MPa and 7.4 MPa, respectively, which is 40% and 11% lower than that of the SP basic system.

The flexural strength of SPB and SPF composite systems was increased using quicklime. The 8 h and 28 d flexural strength of the SPCB30 composite system increased by 61% and 13%, respectively, and the 8 h and 28 d flexural strength of the SPCF9 composite system increased by 43% and 13%, respectively. Quicklime improves the stability of ettringite, and hydrated calcium silicate gel plays a reinforcing role, which enhances flexural strength [38].

### 3.3. The Effect of Quicklime on the Mass Change Rate of SPB and SPF Repair Mortar under Sulphate Attack

**Figure 5:**
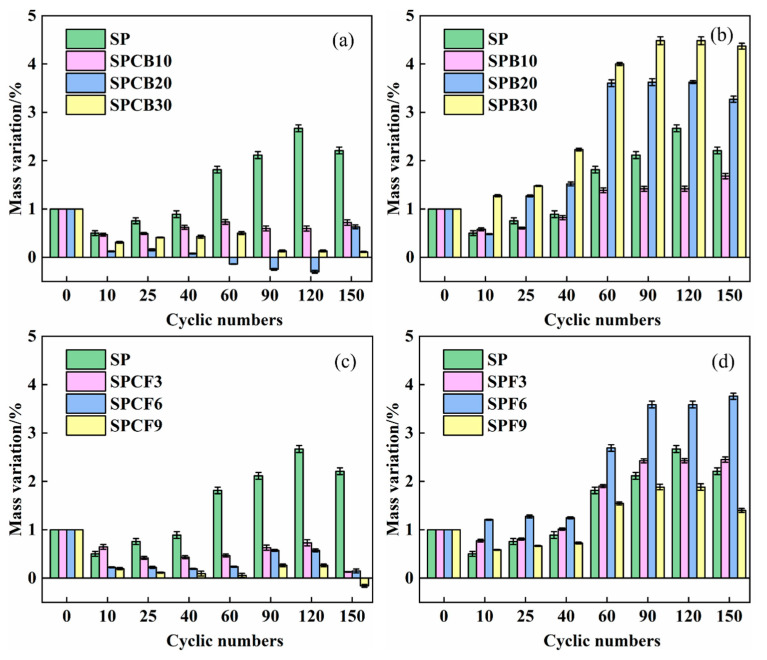
Effect of quicklime on quality change in composite system: (**a**) SPCB; (**b**) SPB; (**c**) SPCF; (**d**) SPF.

Figure 5 exhibits the mass change in SPB and SPF soaked in 5% Na_2_SO_4_ solution under the action of the dry–wet cycle according to Equation (1). As can be seen from Figure 5 that in SPB and SPF composite systems, the mass change rate of a variety of composite systems gradually increases and then tends to be stable as the number of dry–wet cycles rises. With the increase in the content of GGBS and SF, the mass change rate of the specimens increases gradually. Compared to the basic group SP, the mass change in the SPB10 and SPF6 composite systems is lower. The mass change rate of SPB30 is the largest after 150 dry–wet cycles, and the mass growth rate is 4.37%. The mass change rate of SPF9 is the smallest after 150 dry–wet cycles, with a 1.4% mass growth rate.

After the addition of quicklime, the mass change rate of the composite system was greatly reduced, and the mass change rate of the SPCB30 and SPCF9 composite systems decreased to 0.11% and −0.76% after 150 dry–wet cycles. It points to the fact that the addition of quicklime slows down the erosion and expansion of sulphate. In the early stage of the sulphate dry–wet cycle, the continuous hydration of cement along with the intrusion of sulphate ions into the internal pores of the specimen and the hydration products of cement hydrated to form ettringite, which causes the quality of the specimen to continuously improve. In the later stage, it tended to be stable. The erosion products are gathered to fill the internal pores of the mortar to make the mortar dense. After adding quicklime, the pozzolanic activity of the mineral admixture of the composite system was stimulated, and the free water in the slurry was reduced when the quicklime was digested. This decreased the porosity and improved the microstructure of the composite system, with small quality changes [39,40]. Two major mechanisms are identified for the improvements: firstly, the addition of quicklime reduces the porosity and refines the pore structure (before the sulphate attack), which then limits the ingress of sulphate ions into the mortars during the sulphate attack; and secondly, the consumption of available portlandite from the pozzolanic reaction of silica fume and the ground granulated blast furnace slag reduces the amount of forming expansive products, such as gypsum and secondary ettringite [41,42].

### 3.4. Effect of Quicklime on Mechanical Properties of SPB and SPF Repair Mortar under Sulphate Attack

**Figure 6:**
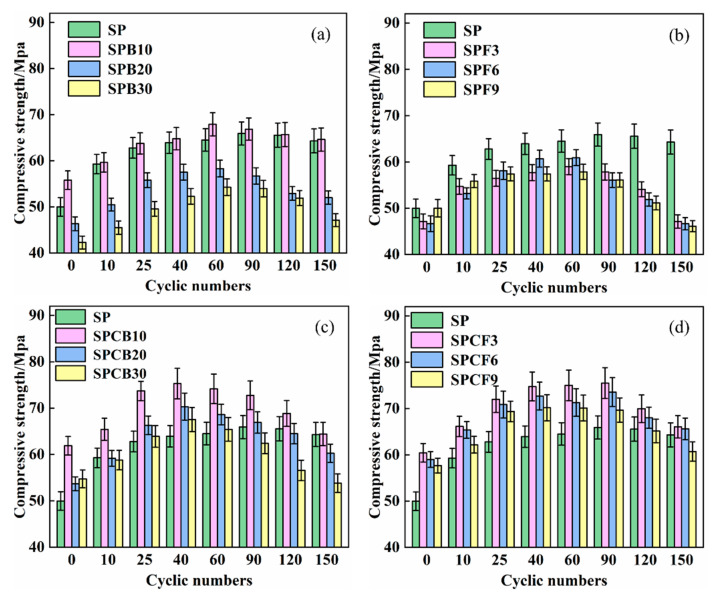
Compressive strength changes of different composite systems:(**a**) SPB; (**b**) SPF; (**c**) SPCB; (**d**) SPCF.

The change in compressive strength of SPB and SPF soaked in 5% Na_2_SO_4_ solution following 150 dry–wet cycles with different GGBS and SF content is displayed in Figure 6. According to Figure 6, the compressive strength of the composite system increases initially before decreasing as the dry–wet cycle erosion time increase. The compressive strength of SPB and SPF composite systems gradually decreases with the rise of GGBS and SF content. The compressive strength of the SPB composite system reached its maximum average value of 60.2 MPa after 60 dry–wet cycles, with an average increase of 25.3% compared to the initial value. The compressive strength began to decrease after 60 dry–wet cycles and decreased by 9.6% on average after 150 dry–wet cycles. The compressive strength of the SPB composite system decreased the fastest with the addition of 30% GGBS. The SPF composite system reaches its maximum strength of 59.3 MPa after 60 dry–wet cycles, with an average increase of 23.8% in compressive strength. After 150 cycles, the compressive strength of the SPF composite system decreases by an average of 21.3%. It indicates that the sulphate corrosion resistance of the sample mixed with GGBS in the basic system is better than that of the SF.

After the addition of quicklime, the SPCB and SBCF composite system maintained high strength throughout the entire test cycle. After 40 dry–wet cycles, the compressive strength of the SPCB composite system reached its maximum value of 71.0 MPa, with an average increase of 25.4% in compressive strength. The SPCB composite system decreased by an average of 16.4% after 150 dry–wet cycles. The maximum strength of the SPCF3 and SPCF6 composite system reached 74.5 MPa after 90 dry–wet cycles, with an average increase of 24.8% in compressive strength. The average decrease in compressive strength after 150 dry–wet cycles was 11.7%. The SPCF9 composite system reached its peak of 70.2 MPa after 40 dry wet cycles, with an increase of 23.8% in compressive strength. After 150 dry–wet cycles, the strength decreased by 12.3%. The compressive strength of the composite system composed of quicklime and SF has the smallest decrease, and the sulphate corrosion performance of the composite system has been significantly improved. The higher volcanic ash reactivity of silica fume results in a denser cement mortar structure and retards the erosion of sodium sulphate [43]. Ca^2+^ improves the strength development and pore structure of mortar, reduces the dispersion of carbonate ions to a certain extent, and enhances the resistance to sulphate erosion [44]. Ca^2+^ participates in the volcanic ash reaction of silica fume, generating hydrated calcium silicate and hydrated aluminic acid with gelling properties to promote the conversion of hydration products into more stable and high-strength hydration products, and the resulting crystals and gels can fill the internal voids, improve the compactness, and improve the resistance of the matrix to sulphate erosion [45].

**Figure 7:**
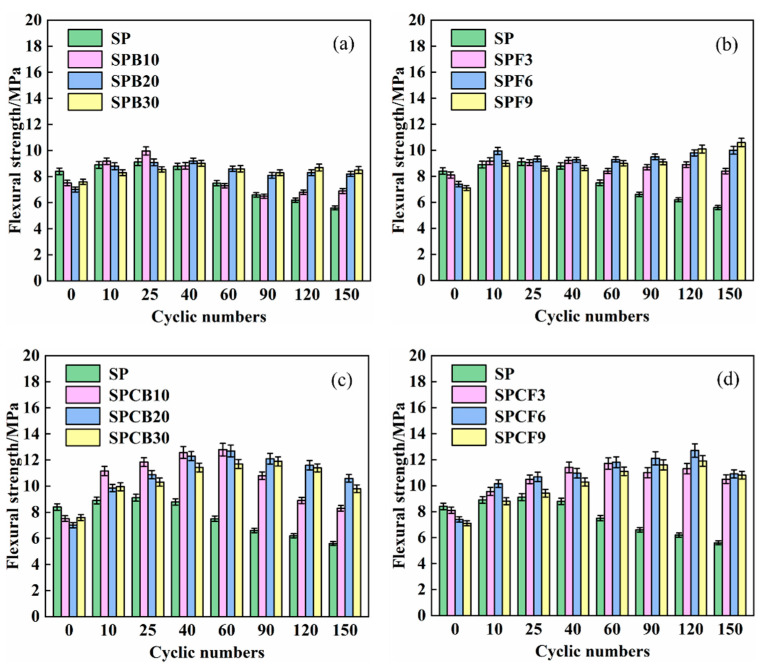
The flexural strength changes of different composite systems: (**a**) SPB; (**b**) SPF; (**c**) SPCB; (**d**) SPCF.

The flexural strength of SPB and SPF immersed in 5% Na_2_SO_4_ solution after 150 dry–wet cycles with varying GGBS and SF content following the addition of quicklime is indicated in Figure 7. The flexural strength of the SP system, as revealed in Figure 7, decreases with the increase in dry–wet cycles. The flexural strength achieves its highest value of 9.1 MPa after 25 dry–wet cycles, and the flexural strength decreases to 5.6 MPa after 150 dry–wet cycles.

Compared with the SP composite system, the addition of GGBS and SF reduces the amplitude of change in the flexural strength of the composite system, making the flexural strength of different composite systems tend to stabilize with the increase of dry–wet cycles. The flexural strength of the SPB composite system decreased by an average of 6.9% compared to its highest value after 150 dry–wet cycles, while the flexural strength of the SPF composite system increased by an average of 29.4% compared to the initial value after 150 dry–wet cycles, indicating that the flexural strength of the composite system increased more after the addition of SF than that of GGBS. After the addition of quicklime, the flexural strength of the SPCB composite system increased by 30.2% after 150 dry–wet cycles, while the flexural strength of the SPCF composite system increased by 43.0% after 150 dry–wet cycles, indicating that the addition of quicklime improved the flexural strength of the SPB and SPF composite systems. Quicklime breaks the Si-O and A1-O bonds in the glassy state of the composite admixture and react with the hydration products Ca (OH)_2_ to produce C-S-H, Aft, and other hydration products, thus improving the flexural strength of the cement. The investigators seem to conclude that the reaction between the amorphous siliceous body of the mineral admixture and hydrated quicklime (towards the formation of additional pozzolanic C-S-H), is mainly responsible for the beneficial action of the flexural strength. This is probably the outcome of the continuous generation of pozzolanic reaction products that fill the pores [46,47].

### 3.5. The Effect of Quicklime on the Phase and Amount of Hydration Products of SPB and SPF Pastes

**Figure 8:**
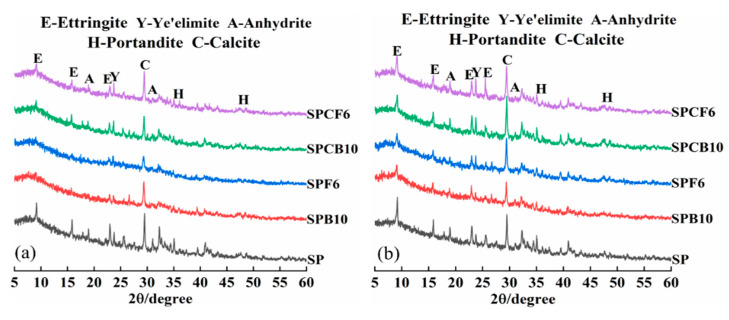
XRD diagrams of different composite systems: (**a**) water; (**b**) sulfate.

Figure 8 is the XRD diagram of different composite systems at 90 days under water curing, as well as the sulphate dry–wet cycle. According to Figure 8, the hydration products of the composite system under the sulphate dry–wet cycle are consistent with the hydration products of water, which primarily consist of ettringite (AFt), anhydrous calcium sulphoaluminate (Ye ‘elimite), calcium hydroxide (CH), calcium aluminosilicate hydrate, and other phases. Figure 7a illustrates that the AFt diffraction peak of the hydration product of the mixed GGBS, and SF is weaker in comparison to that of the basic group, and the diffraction peak of the main component Ye‘elimite of the unhydrated cement is reduced. The main component Ye‘elimite of cement is reduced by the addition of GGBS and SF, and the alkalinity of the composite system is insufficient. Therefore, less AFt will form in the composite system.

The AFt diffraction peak was improved after quicklime was added, indicating that quicklime accelerated the hydration rate of GGBS and SF, and AFt crystal was formed. After the dry–wet cycle of sulphate, the AFt peak and calcium carbonate peak of the SPF6 composite system were dramatically enhanced. After adding quicklime, the GGBS and SF in the composite system consumed Ca(OH)_2_, and the AFt diffraction peak of the sulphate dry–wet cycle was lower as compared to that of the SP base group, and this points out that the mineral admixture was well matched with quicklime, which may delay the erosion of sulphate ions while lowering the number of expansion products generated by a chemical reaction between sulphate and hydration products. Because the sulphate attack of the composite system is primarily the filling stage, the expansion stage has not yet occurred, the reaction products in the pores are filled with pores, and no expansion failure occurs.

**Figure 9:**
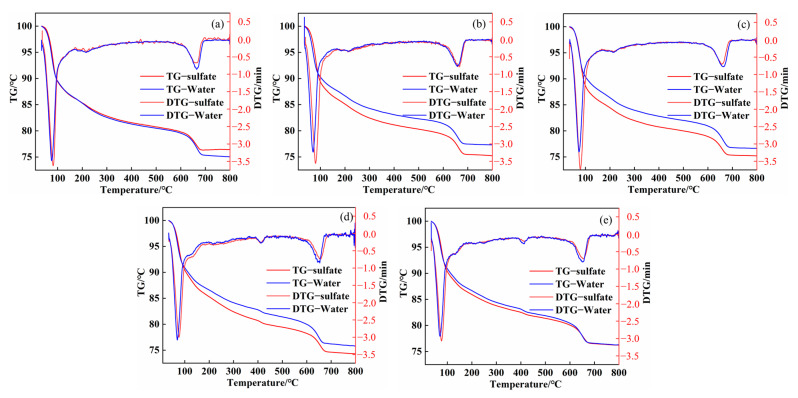
Thermogravimetric diagram of different cement pastes: (**a**) SP; (**b**) SPB10; (**c**) SPF6; (**d**) SPCB10; and (**e**) SPCF6.

The TG-DTG diagram of a variety of composite systems from room temperature to 800 °C, following a dry–wet cycle with sulphate in water, can be found in Figure 9. As can be observed in Figure 9, there are several obvious endothermic peaks in the DTG curve. The endothermic peaks at 57.2–107.3 °C are AFt and C-S-H gel, the endothermic peak at 619.8–798.9 °C is CaCO_3_, whereas the endothermic peak at about 400 °C is Ca (OH)_2_. The lack of an endothermic calcium hydroxide peak in clear water in the SP system suggests that the calcium hydroxide created in the composite system is not very abundant. The mass losses of AFt, C-S-H gel, and CaCO_3_ in the SP basic system are 17.08% and 6.22%, respectively. After adding mineral powder and silica fume, the mass loss of AFt, C-S-H gel, and CaCO_3_ is reduced to 16.29%, 6.01% and 16.96%, and 6.16%, highlighting that the incorporation of mineral admixtures reduces the hydration products, which is in line with the previous XRD results.

After adding quicklime, the mass loss of AFt, C-S-H gel, and CaCO_3_ of SPCB10 and SPCF6 specimens were 16.78%, 7.06% and 16.30%, and 7.11%, respectively. The mixture of quicklime and mineral powder increased the amount of AFt and C-S-H gel, and the calcium carbonate crystals formed by the reaction of lime increased. This improved the pore structure of the repair material system and was advantageous to the mechanical properties. After the sulphate dry–wet cycle, the mass loss of AFt crystal and C-S-H gel in different composite systems dramatically rose, whereas the mass loss of CaCO_3_ decreased. The mass losses of AFt, C-S-H gel, and CaCO_3_ in the SP basic system were 18.36% and 4.93%. The mass losses of AFt, C-S-H gel, and CaCO_3_ in the composite system containing GGBS and SF were 18.61%, 5.81% and 18.87%, and 6.39%. This finding implies that the incorporation of GGBS and SF in the sulphate environment will make the composite system produce more AFt and C-S-H gel. When the quicklime is added, the mass losses of AFt and C-S-H gel in SPCB10 and SPCF6 composite systems was 18.77%, 5.50% and 18.24%, and 6.31%, respectively. As compared to SPB10 and SPF6, the mass loss of CaCO_3_ was lower. The mass loss of AFt and C-S-H gel in the SPCB10 composite system increased, while the mass loss of AFt and C-S-H gel in the SPCF6 composite system decreased. Under alkaline conditions, mineral admixtures in combination with Ca^2+^ produced by quicklime form C-S-H gel, and the remaining Ca^2+^ reacts with CO_3_^2−^ to form CaCO_3_ [48,49].

### 3.6. Effect of Quicklime on Microstructure and Pore Characteristics of SPB and SPF Hydration Products

**Figure 10:**
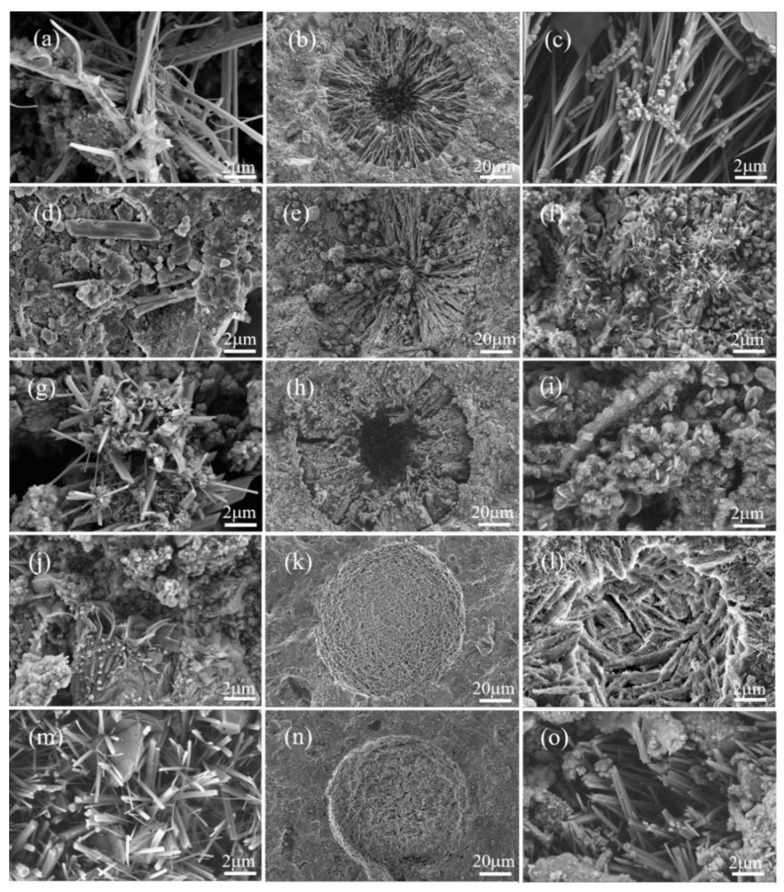
Microstructures of different cement pastes: water 2 μm: (**a**) SP; (**d**) SPB10; (**g**) SPF6; (**j**) SPCB10; (**m**) SPCF6. Sulphate 20 μm: (**b**) SP; (**e**) SPB10; (**h**) SPF6; (**k**) SPCB10; (**n**) SPCF6. Sulphate 2 μm: (**c**) SP; (**f**) SPB10; (**i**) SPF6; (**l**) SPCB10; (**o**) SPCF6.

The microscopic morphology of the composite system under the action of clear water 2 μm and sulphate erosion 20 μm and 2 μm is depicted in Figure 10. The clear water makes it evident that the SP base system of the composite system, where the majority of the needle-like AFt is created, has pores on the surface. After adding GGBS and SF, C-S-H gel was formed, which became dense and less porous compared with the basic system. Figure 9j,m show that the pozzolanic activity of GGBS and SF is effectively stimulated when the quicklime was added, and a large amount of hydrated calcium silicate gel is produced by the hydration reaction of GGBS and SF. The ettringite crystal and hydrated calcium silicate gel are intertwined and aggregated in the system and are continuously filled in the pores, making the structure of the hydration product more compact and enhancing the mechanical properties of the composite system [50,51].

Under the action of sulphate erosion, there are micro cracks in the basic system, and ettringite becomes slender and needle-like. Figure 10b,c are the microscopic morphology of SP composite system after dry–wet cycles. The macropores of SP composite system are filled with needle-shaped directional AFt, whose direction is from the hole wall to the hole center, and the generation of internal microcracks can also be observed. During the dry–wet cycles, SO_4_^2−^ enters the interior of the test piece from the solution, reacts with C_3_A, and generates ettringite. With the increase in dry–wet cycles, ettringite with expansion continues to accumulate. When the expansion stress is greater than the tensile strength inside the concrete, new cracks will appear inside, and micro cracks will gradually develop, ultimately leading to concrete cracking and failure. Therefore, as the number of dry–wet cycles increases, the flexural strength of the SP composite system shows a decreasing trend.

After the addition of GGBS and SF, an increasing amount of gel and AFm were generated. Figure 10e,f show that the SPB10 composite system mixed with GGBS generates a large amount of gel to cover the surface, no cracks are observed, and the microstructure becomes more compact. Therefore, with the increase in the number of dry–wet cycles, the compressive strength of the SPB10 composite system is higher than that of the basic SP system, showing an increasing trend. The addition of SF results in a porous and porous SPF6 composite system compared to the SPB10 composite system. After the addition of quicklime, the ettringite of the composite system became coarse and wrapped between the C-S-H gel, making the entire composite system relatively dense. The aluminum hydration products in the cement matrix react with the small amount of intruding sulphate ions to form an expansive AFt that fills the pores and cracks within the concrete. The quicklime reduces the solubility of calcium alumina and facilitates the stable presence of calcium alumina [52]. According to their mechanical properties, SPCB10 and SPCF6 have good sulphate erosion performance.

**Figure 11:**
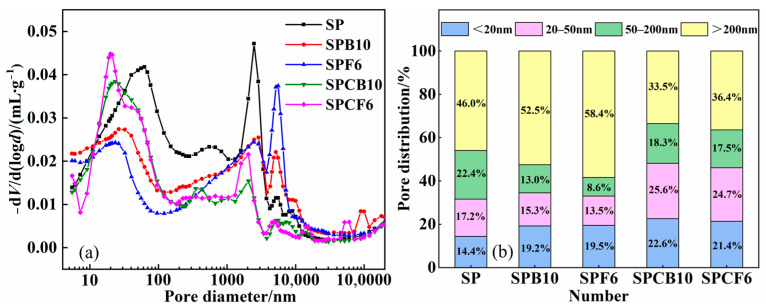
Pore volume maps of different repair mortars: (**a**) pore size distribution map; (**b**) pore size distribution histogram.

The pore size distribution, as well as the pore size distribution histogram of different repair mortar composite systems at 28 d, are shown in Figure 11. The maximum porosity of the basic SP cement mortar is 19.93%, as can be observed in Figure 11. The porosity drops to 18.4% and 15.1% after 10% GGBS and 6% SF are added, respectively. The mineral admixture has a large specific surface area, which lowers the mortar’s internal porosity.

The porosity of the composite system was reduced by adding quicklime, and the porosity of SPCB10 and SPCF6 was 15.54% and 14.62%. It can be seen from Figure 11b that the diameter of the pores of the test block has changed greatly after adding quicklime, while the number of pores with relatively small particle sizes has increased rapidly. Combined with the microstructure analysis, the appearance of ettringite and hydrated calcium silicate will alter the distribution of small pores inside the test block to some degree, which will then have an impact on the strength change in the test block. In contrast to the porosity of larger pore size, it is further reduced.

## 4. Conclusions

(1) Adding quicklime prolongs the setting time of the composite system. The final setting time is 5 min and 9 min longer than that of SPB and SPF, respectively.

(2) The early strength of the composite system was dramatically improved after the addition of quicklime. The 8 h compressive strength of the SPCB30 and SPCF9 composite system improved by 191% and 120%, respectively. The 8 h flexural strength of the SPCB30 and SPCF9 composite system increased by 61% and 43%, respectively.

(3) After the quicklime was added, the formation of C-S-H gel and calcium carbonate in SPB and SPF composite systems was promoted, the porosity was reduced, and the pore structure was refined. The porosity was reduced by 2.68% and 0.48%, respectively. The mass change rate of various composite systems under sulphate attack was reduced, and the mass change rate of the SPCB30 and SPCF9 composite systems decreased to 0.11% and −0.76% after 150 dry–wet cycles. and the mechanical strength of different composite systems under sulphate attack was improved, so that the sulphate resistance of different ground granulated blast furnace slag and silica fume composite systems was improved.

(4) In addition to stimulating the activity of mineral admixtures to yield more ettringite and C-S-H gel in SPCB and SPCF, quicklime also increases the system’s alkalinity and the stability of AFt. For sulphate repair materials, the sulphate corrosion resistance can be improved by including mineral admixtures and a small amount of quicklime.

The current study only focuses on effect of sulphate attack resistance of repair mortars. However, it should be noted that the bonding of the old and new interfaces is one of the keys. The anti-sulphate erosion performance of the interface of the composite system under sulphate erosion environment should be further studied, and the relationship between the interfacial microstructure and the interfacial bonding performance should be investigated to reveal the evolution of the interfacial microstructure under sulphate environment and the mechanism of the degradation of the interfacial bonding performance caused by it, which can better guide the actual application of the repair material.

## Figures and Tables

**Table 1:** Chemical composition of cement and mineral admixtures/wt.%.

Oxide	CaO	Al_2_O_3_	SiO_2_	Fe_2_O_3_	MgO	SO_3_	K_2_O	Na_2_O
OPC	63.28	5.81	19.72	4.36	1.95	2.63	0.18	0.29
CSA	42.25	36.46	6.56	2.28	1.84	8.92	0.18	0.24
GGBS	34.00	17.70	34.50	1.03	6.01	1.64	0.56	0.34
SF	0.71	0.54	96.00	0.65	0.68	0.18	0.40	0.17

**Table 2:** Properties of cement.

Cement	Setting Time (min)		Flexural Strength (MPa)		Compressive Strength (MPa)		Secific Surface Area (m^2^/kg)	Secific Gravity (g/cm^3^)
	Initial Set	Final Set	3 d	28 d	3 d	28 d		
OPC	63	115	6.1	7.8	29.4	49.6	350	3.1
CSA	16	26	6.5	8.2	36.5	50.8	428	2.9

**Table 3:** Test mix ratio of repair mortar.

Sample	CSA/g	OPC/g	GGBS/g	SF/g	Quicklime/g
SP	490.0	210.0	0.0	0.0	0.0
SPCB10	441.0	189.0	70.0	0.0	35.0
SPCB20	392.0	168.0	140.0	0.0	35.0
SPCB30	343.0	147.0	210.0	0.0	35.0
SPB10	441.0	189.0	70.0	0.0	0.0
SPB20	392.0	168.0	140.0	0.0	0.0
SPB30	343.0	147.0	210.0	0.0	0.0
SPCF3	475.3	203.7	0.0	21.0	35.0
SPCF6	460.6	197.4	0.0	42.0	35.0
SPCF9	445.9	191.1	0.0	63.0	35.0
SPF3	475.3	203.7	0.0	21.0	0.0
SPF6	460.6	197.4	0.0	42.0	0.0
SPF9	445.9	191.1	0.0	63.0	0.0

## Data Availability

The data presented in this study are available on request from the corresponding author.
